# Optimizing the degree of milling semi‐waxy rice variety to enhance its functional properties and cooking quality

**DOI:** 10.1111/1750-3841.70142

**Published:** 2025-03-20

**Authors:** Youngho Kwon, Gi‐Un Seong, Ju‐Won Kang, So‐Myeong Lee, Jin‐Kyung Cha, Hyunjin Park, Byungjun Jin, Youngeun Lee, Su‐Min Jo, Woo‐Jae Kim, Seong‐Gyu Jang, Jun‐Hyeon Cho, Dong‐Soo Park, Jong‐Hee Lee

**Affiliations:** ^1^ Department of Southern Area Crop Science, National Institute of Crop Science Rural Development Administration Miryang Republic of Korea; ^2^ Food Safety and Distribution Research Group Korea Food Research Institute Wanju‐gun Republic of Korea

**Keywords:** bioactive compounds, milling optimization, semi‐waxy rice

## Abstract

**Abstract:**

Rice is a staple food worldwide, yet its nutritional quality is considerably impacted by the milling process, which removes the bran layer and other outer parts containing bioactive compounds. This study aimed to optimize the milling degree of the semi‐waxy rice variety “Milyang 387” (M387) to retain key functional components, such as γ‐aminobutyric acid (GABA) and γ‐oryzanol, while preserving desirable cooking qualities. The study compared the antioxidant capacities, including GABA and γ‐oryzanol content, and physicochemical properties, such as amylose content and texture, at varying milling degrees between “Nampyeong” (intermediate) and “M387” (semi‐waxy) rice varieties. Results show that milling significantly influenced the bioactive compound content within each variety, with higher degrees of milling leading to reduced bioactive retention. This confirms the effect of milling on nutrient retention rather than varietal differences. A milling degree of approximately 9.5% was identified as optimal, balancing bioactive compound retention with texture characteristics for semi‐waxy variety “M387.” “M387” exhibited superior antioxidant capacity and cooking quality at this milling degree, making it a promising candidate for both health‐conscious consumers and those seeking high‐quality, esthetically pleasing rice. The findings of this study provide a valuable framework for optimizing rice milling practices to retain bioactive compounds while ensuring desirable cooking quality. The optimal milling degree of 9.5% was determined based on its ability to retain higher levels of bioactive compounds compared to higher milling degrees while ensuring desirable cooking texture. These results highlight controlled milling processes in enhancing the nutritional value of rice, supporting the development of processing strategies that maximize health benefits.

**Highlights:**

- Identifying the optimal milling degree of semi‐waxy rice “M387” at 9.5%, achieving a balance between bioactive compound retention and desirable cooking properties. Providing insights into optimizing milling practices to enhance nutritional value, functional properties, and consumer acceptability of semi‐waxy rice varieties.

## 1 INTRODUCTION

Grains, including rice, wheat, legumes, and their processed derivatives, such as flour and starch, serve as the cornerstone of global food systems, contributing significantly to both caloric intake and nutritional health. In 2021, global grain production exceeded 418 million tons, underscoring their indispensable role in feeding the world's population (FAO, 2022). Among these crops, rice (*Oryza sativa* L.) is particularly notable, ranking as the principal staple food for more than half of the world's population. Its demand is predicted to increase by 40% in 2040 (Seck et al., 2012). Beyond its value as an energy source, rice is increasingly appreciated for its physiological and functional properties, which have become the subject of extensive nutritional research (Goufo & Trindade, 2014; Gul et al., 2015; Pascual et al., 2013). Supporting information

Rice processing, especially the degree of milling (DOM), is a critical factor influencing both its nutritional content and consumer acceptance. The milling process involves removing the bran layer, germ, and other outer layers of the rice kernel, which are rich in dietary fiber, vitamins, minerals, and bioactive compounds (Cho & Lim, 2016; Mir et al., 2020; Tan et al., 2023). While consumers prefer polished white rice for its clean appearance and soft texture, this preference comes at the cost of reduced nutritional value (Choi et al., 2018; Mir et al., 2020; Ojediran et al., 2020; Yi et al., 2024). The bran layer, which is removed during milling, contains various physiologically active substances, including γ‐aminobutyric acid (GABA), γ‐oryzanol, dietary fiber, and essential minerals such as calcium and iron (Gul et al., 2015; Manzoor et al., 2023; Park et al., 2017; Sharif et al., 2014). These components are crucial for health promotion, providing benefits such as improved digestion, enhanced cardiovascular function, and neuroprotection (Borresen & Ryan, 2014; Sohail et al., 2017).

Among the key bioactive compounds found in rice, GABA stands out for its significant role as a neurotransmitter in the central nervous system, where it aids in regulating mood, promoting relaxation, and enhancing cognitive function (Li et al., 2021). GABA is particularly abundant in the bran layer of unpolished rice and certain semi‐waxy varieties, but its levels are drastically reduced during milling. Previous studies have shown that GABA‐rich diets can contribute to improved memory, reduced anxiety, and even lower blood pressure (Kalueff & Nutt, 2007). Similarly, γ‐oryzanol, another important bioactive compound, has been linked to various health benefits, including anti‐inflammatory, cholesterol‐lowering, and anticarcinogenic properties (Sawada et al., 2019). Similar to GABA, γ‐oryzanol is predominantly concentrated in the outer layers of the rice grain, making it vulnerable to degradation during polishing (Chang et al., 2023).

Despite these well‐documented benefits, conflict persists between the nutritional optimization of rice and the preferences of consumers, who typically favor highly polished, esthetically pleasing rice. Polished rice, though visually appealing, is often devoid of essential nutrients and bioactive compounds, rendering it less beneficial from a health perspective (Liu et al., 2015; Mir et al., 2020). This dichotomy highlights the need for optimized milling practices that balance the retention of key nutrients with the maintenance of desirable sensory qualities such as texture, color, and palatability.

In the context of semi‐waxy rice varieties, characterized by lower amylose content and a softer, stickier texture, the challenge of optimizing milling practices becomes even more pronounced. Semi‐waxy varieties, such as “M387,” are preferred for certain culinary applications, such as sushi or rice balls, where a softer texture is desired. However, the milling process, particularly when conducted to higher degrees, can strip away valuable components such as GABA, γ‐oryzanol, and other antioxidants, which play critical roles in human health (Chang et al., 2023; Pascual et al., 2013; Sharif et al., 2014). Studies have shown that optimizing the milling degree can help retain these bioactive compounds while achieving acceptable cooking and sensory qualities (Yi et al., 2024).

This study evaluated the semi‐waxy rice variety “M387” under five different milling degrees (5.5%, 7.5%, 9.5%, 11.5%, and 13.5%) to assess changes in antioxidant capacity and physicochemical properties. For comparison, the Japonica rice variety “Nampyeong” was showed lower antioxidant capacity and physicochemical properties than “M387” under the same milling treatments. The primary aim is to determine the optimal milling degree that maximizes the retention of GABA and γ‐oryzanol while maintaining desirable cooking qualities, such as texture and taste. By analyzing antioxidant activity and functional component retention across different milling degrees, this study aims to contribute to the broader understanding of how milling practices can be optimized for both health benefits. Current research lacks a comprehensive understanding of how varying milling degrees influence the retention of bioactive compounds (e.g., GABA and γ‐oryzanol) and cooking quality specific to these varieties. Ultimately, the findings of this research may help guide future efforts in rice processing, offering a balanced approach that enhances the nutritional value of rice without compromising on quality.

## 2 MATERIALS AND METHODS

### 2.1 Plant materials

The samples used in this study were sourced from “M387” and produced in accordance with the standard cultivation method of the Rural Development Administration. Additionally, “Nampyeong,” a variety selected for comparative analysis, was included. “Nampyeong” is a widely cultivated Japonica rice variety characterized by a medium maturity type. Post‐harvesting, the rice was stored in a paddy state at a low temperature of 15°C. An impeller‐type brown rice milling machine (FC2K) was employed for husk removal, yielding the brown rice selection.

Subsequently, polishing and sample preparation were conducted using a friction‐type rice polisher (VP‐32). Two hundred grams of brown rice were polished at a time. The degree of polishing was precisely set within the range of 5.5%–13.5%. Considering that the weight ratio of brown to white rice was 100%, rice weight would be 94.5%–86.5%. The formula for calculating the DOM is as follows:

$$\mathrm{DOM}\;\left(\%\right)=\frac{\mathrm{Weight}\;\mathrm{of}\;\mathrm{brown}\;\mathrm{rice}\;\left(g\right)-\mathrm{Wieght}\;\mathrm{of}\;\mathrm{polished}\;\mathrm{rice}\;\left(g\right)}{\mathrm{Weight}\;\mathrm{of}\;\mathrm{brown}\;\mathrm{rice}\;\left(g\right)}\;\times 100$$

This approach was adopted to apply the conventional concept of milling degree, wherein the theoretical 12% DOM translated to 90.4% (Goufo & Trindade, 2014; Kim et al., 2020; Liu et al., 2015; Shewry et al., 2023). Expressing this as the degree of polishing would result in 9.5%. The degree of polishing was categorized into five levels (5.5%, 7.5%, 9.5%, 11.5%, and 13.5%), each indicating that the degree of polishing was adjusted incrementally by 2% above or below the reference level of 9.5%. The white rice was further refined and separated from the bran using a bran separator (Model TRG 05A; Satake Engineering Co., Ltd.) equipped with a 3.6‐mm cylindrical sieve for 2 min. To analyze chemical components, the rice flour was ground using a Cyclon mill (Udy Analyzer Co.), followed by sieving through a 100‐mesh sieve. Moreover, the formula for calculating the unstripped embryo rate is as follows:

$$\begin{array}{ccc} & & \mathrm{Unstripped}\;\mathrm{embryo}\;\mathrm{rate}\;=\\ & & \frac{\mathrm{Number}\;\mathrm{of}\;\mathrm{grains}\;\mathrm{with}\;\mathrm{intact}\;\mathrm{embryos}}{\mathrm{Total}\;\mathrm{number}\;\mathrm{of}\;\mathrm{grains}\;\mathrm{observed}}\times 100 \end{array}$$

The embryos were visually observed under controlled lighting conditions to determine their retention on the surface of the milled rice. The unstripped embryo rate was calculated as the percentage of rice grains retaining an intact embryo structure following milling.

For accuracy and consistency, a standardized quantitative assessment was conducted. The polished rice samples were spread evenly on a black background under uniform illumination, and high‐resolution images were captured using a digital imaging system. The images were then analyzed using an automated image processing tool (ImageJ), which classified and counted the grains with visible embryo structures. A manual validation step was performed by trained analysts to confirm the accuracy of the automated counts.

### 2.2 Physicochemical properties

#### 2.2.1 Moisture content

The moisture content of the rice samples was determined using a moisture meter (MS‐70). The instrument uses the principle of measuring the electrical conductivity of a substance to determine its moisture content. The analyses were conducted thrice, and average values are presented.

#### 2.2.2 Amylose content

The amylose content of rice samples was determined using the method described by (Suwannaporn et al., 2007), which involves solubilization and color development. To prepare the sample, 0.1 g of rice flour was mixed with 1 mL of ethanol and 9 mL of 1 N sodium hydroxide solution, and the mixture was then incubated at 90°C for 20 min. The volume was brought up to 100 mL using distilled water, and an aliquot of 5 mL was mixed with 1 mL of 1 N acetic acid and 2 mL of iodine solution (*I*_2_ in 2% KI). Thereafter, the mixture was incubated at 30°C for 20 min, and the absorbance was measured at 620 nm using an ultraviolet/visible spectrometer (UV‐2700; Shimadzu). The amylose concentrations were determined using the amylose standard curve generated from the absorbance at 620 nm.

#### 2.2.3 Crude protein content

The crude protein content of rice samples was determined using near‐infrared spectroscopy (XM‐1100 series; FOSS NIR Systems INC.). To prepare the sample, 0.6 g of rice flour was added to the micro insert ring bound to the mini sample cup, and the air gap in the sample was removed using the sample cup disposable back. NIR spectroscopy was then performed using visible and near‐infrared wavelengths (400–2500 nm) under room temperature conditions (25°C). ISI scan software (version 4.5.0; InfraSoft International) was used to calculate the crude protein content.

#### 2.2.4 Color value

The color of rice samples was measured using a colorimeter (CM‐3500d; Konica Minolta. In addition, to evaluating the L* (lightness), a* (red/green), and b* (yellow/blue) color parameters using the CIELAB system, the whiteness index (WI), hue angle (*h*°), and saturation (C*) were calculated to provide a more comprehensive assessment of the color attributes. WI was determined using the formula $=100-\sqrt{{\left(100-L*\right)}^{2}+a^{2}+b^{2}}$.

#### 2.2.5 Toyo taste value

The Toyo taste meter (MA‐90A and 90B; Toyo Co.) is a widely used and reliable instrument for assessing the mechanical palatability of rice. The instrument features an automated cooking part, which ensures consistent cooking conditions for all samples. To perform the measurement, 33 g of rice were placed in the cooking part and cooked for 10 min. The cooked rice was then allowed to rest at room temperature (approximately 23°C) for 2 min before the Toyo taste value was measured. Toyo taste value measurement provides a quantitative assessment of the mechanical palatability of rice and is a valuable tool for evaluating rice quality.

#### 2.2.6 Texture analysis

The hardness and adhesiveness of the rice samples were determined using a hardness‐stickiness texture analyzer (RHS1A; SATAKE Co. Ltd.). Rice samples (30 g) were placed in an aluminum container and washed with water until the water became clear. Next, the samples were left to soak for 30 min, after which water with a final mass of 42 g was added to the aluminum container. The rice samples were cooked in an electronic steam cooker (Model RHS‐1A; SATAKE, Hiroshima, Japan) for 30 min. After cooking, the samples were cooled for 20 min to reach a final temperature of approximately 23°C, which is suitable for texture analysis. The cooled rice was placed in a circular steel ring of approximately 4‐cm diameter and 1‐cm width and compacted before measurement using the hardness‐stickiness texture analyzer.

#### 2.2.7 Rapid visco analysis

The pasting properties of rice samples were analyzed using a rapid viscosity analyzer (RVA‐4500; Perten Instruments). To perform the analysis, 3.0 g of rice flour with a 12% moisture content was added to an aluminum container, followed by 25 mL of distilled water. Next, the samples were incubated at 50°C for 1 min, heated to 95°C for 3.45 min, held at 95°C for 2.7 min, cooled to 50°C for 3.91 min, and maintained at 50°C for 1.24 min. The following parameters were recorded and analyzed using the instrument: peak, breakdown, and setback viscosity.

### 2.3 Antioxidant capacity

#### 2.3.1 Ferulic acid content

The ferulic acid content was determined using a previously described method (Zhou et al., 2004) with minor modifications. Zhou et al. (2004) provided the foundational method for ferulic acid extraction and quantification using high‐performance liquid chromatography (HPLC). Recognizing the dense bran structure of “M387,” which may hinder efficient compound release, this study extended the sonication duration from 10 to 20 min. This modification considerably improved extraction efficiency, allowing for a more comprehensive analysis of ferulic acid content in minimally processed rice samples. Briefly, 200 mL ethanol was added to the samples (10 g), which were extracted by reflux cooling for 3 h. Thereafter, the extract was concentrated using a vacuum concentrator, and 30 mg of the concentrated sample was dissolved in ethanol to a concentration of 10 mg/mL. Next, the solution was sonicated for 20 min and filtered through a 0.2‐µm PVDF membrane filter to obtain the test solution. Subsequently, the ferulic acid content was analyzed using HPLC (Waters Alliance 2695 HPLC system with a 996 PDA detector [Waters Corporation]) with an INNO C18 column (250 × 4.6 mm^2^, 5 µm; [Young Jin Biochrom Co., Ltd.]) and UV—visible detector at 324 nm. The following binary mobile phases were used: A, distilled water; B, acetonitrile. The gradient for HPLC analysis was linearly conducted for a total of 50 min as follows: 90% A/10% B at 0 min, 90% A/10% B at 5 min, 70% A/30% B at 20 min, 50% A/50% B at 25 min, 0% A/100% B at 30 min, 0% A/100% B at 35 min, 90% A/10% B at 40 min, and 90% A/10% B at 50 min. The flow rate was set to 1.0 mL/min with a column temperature of 35°C. The ferulic acid content of the samples was confirmed and calculated by comparing their individual retention times and peak areas with those of ferulic acid.

#### 2.3.2 Gamma‐oryzanol content

The γ‐oryzanol content was determined using a previously reported method (Lilitchan et al., 2008), with minor modifications. This quantification method was refined to increase throughput without compromising precision. Adjustments to the HPLC gradient program reduced the runtime by 10%, optimizing the analysis of the numerous samples generated from various milling degrees. This tailored approach was particularly beneficial in managing the high volume of data while maintaining accurate γ‐oryzanol measurement. Briefly, samples (20 g) with 400 mL ethanol added were extracted by reflux cooling for 3 h. Afterward, the extract was concentrated using a vacuum concentrator, and 30mg of the concentrated sample was dissolved in ethanol to a concentration of 15 mg/mL. The solution was sonicated for 20 min and then filtered through a 0.45‐µm PVDF membrane filter to obtain the test solution. The γ‐oryzanol content was analyzed using an HPLC (Waters Alliance 2695 HPLC system with a 2489 UV/Vis detector [Waters Corporation]) with a YMC pack pro C18 column (250 × 4.6 mm^2^, 5 µm; [YMC Co., Ltd.]) and UV–visible detector at 325 nm. The following binary mobile phases were used: A, methanol; B, acetonitrile. The gradient for HPLC analysis was linearly conducted for a total of 45 min as follows: 50% A/50% B at 0 min, 65% A/35% B at 10 min, 100% A/0% B at 20 min, 45% A/55% B at 25 min, 50% A/50% B at 30 min, and 50% A/50% B at 45 min. The flow rate was set to 1.0 mL/min with a column temperature of 35°C. The γ‐oryzanol content of the samples was confirmed and calculated by comparing their individual retention times and peak areas with those of γ‐oryzanol, including γ‐oryzanol, oryzanol A, and oryzanol C.

#### 2.3.3 DPPH assay

The DPPH radical scavenging activity of the samples was determined following a previous report (Sharma & Bhat, 2009), with some modifications. The antioxidant capacity was assessed using standard DPPH and ABTS assays, as described by Sharma and Bhat (2009) and Verrillo et al. (2025), respectively. However, reaction times were extended by 10 min to ensure complete interaction between the radical species and the antioxidant compounds. This adjustment was particularly critical for semi‐waxy rice extracts, which exhibited slower diffusion rates due to their unique composition and viscosity. Rice flour used for mixing with the DPPH solution was prepared by grinding white rice samples obtained at different milling degrees using a Cyclon mill (Udy Analyzer Co.) and sieving through a 100‐mesh sieve to ensure uniform particle size. Briefly, 50 µL of the analytical sample was mixed with 200 µL of 0.15 mm DPPH solution in a 96‐well plate and allowed to react in the dark for 30 min. The absorbance was then measured at 517 nm using a multimode plate reader (Thermo Scientific Varioskan Flash; Thermo Fisher Scientific). Results are expressed as gallic acid equivalents (mg GAE/100 g) using the regression equation of the gallic acid standard curve.

#### 2.3.4 ABTS assay

The ABTS radical scavenging activity of the analytical samples, prepared by grinding white rice obtained at different milling degrees using a Cyclon mill, was determined following a previously reported method (Verrillo et al., 2025) with some modifications, similar to those applied in the DPPH assay. Briefly, the ABTS solution was prepared by mixing 7 mM 2,2′‐azino‐*bis* (3‐ethylbenzothiazoline‐6‐sulfonic acid) diammonium salt and 2.45 mM potassium persulfate in a 1:1 (v/v) ratio and allowing the mixture to react for 4h at 4°C. Next, 225 µL of diluted ABTS solution and 25 µL of the analytical sample were added to a 96 well plate, and the mixture was allowed to react for 1 min in the dark. The absorbance was then measured at 734 nm using a multimode plate reader. The results are expressed as Trolox equivalent (mg TE/100 g) by conversion using the regression equation of the Trolox standard curve.

#### 2.3.5 Total phenolic content

The total phenolic content was measured using the Folin−Ciocalteu method. Briefly, 10 µL of the analytical samples, prepared by grinding white rice obtained at different milling degrees using a Cyclon mill, were mixed with 200 µL of 10% sodium carbonate and incubated at room temperature for 3 min. Afterward, 10 µL of 1 N Folin–Ciocalteu phenol reagent was added and incubated for 27 min at 37°C. The absorbance was then measured at 750 nm using a multimode plate reader. The total phenolic content is expressed as gallic acid equivalents (mg GAE/100 g) by conversion using the regression equation of the gallic acid standard curve.

#### 2.3.6 Total flavonoid content

The total flavonoid content was measured using a modified version of a previously reported method (Pękal & Pyrzynska, 2014). The modification involved the aluminum chloride colorimetric assay, which relies on the formation of a stable complex between aluminum ions and flavonoid compounds. In this study, the reaction time for complexation was increased from 10 to 12 min to account for slower reaction kinetics observed in rice extracts. This adjustment ensured complete interaction between aluminum ions and flavonoids, yielding stable and consistent absorbance measurements at 510 nm. Briefly, 70 µL of the analytical samples, prepared by grinding white rice obtained at different milling degrees using a Cyclon mill, were mixed with 430 µL of 50% ethanol and 50 µL of 5% sodium nitrite and allowed to react at room temperature for 20 min. Subsequently, 50 µL of 10% aluminum nitrate nonahydrate was added and allowed to react for 6 min. Next, 500 µL of 1 N sodium hydroxide was added, and the absorbance was measured at 510 nm using a multimode plate reader. The flavonoid content is expressed as catechin equivalents (mg CE/100 g) by conversion using the regression equation of the catechin standard curve.

### 2.4 Statistical analysis

A two‐way analysis of variance (ANOVA) was conducted to evaluate the effects of rice variety (“M387” and “Nampyeong”) and milling degree (5.5%, 7.5%, 9.5%, 11.5%, and 13.5%) on the measured parameters. This approach allowed for the assessment of both main effects and the interaction between variety and milling degree. Each milling treatment was replicated three times, with 200 g of brown rice per replicate polished under the specified conditions. Statistical significance was determined at a *p*‐value of <0.05, and post hoc comparisons were conducted using *t*‐test to identify differences between “M387” and “Nampyeong.” The analyses were performed using SAS Enterprise Guide (7.1; SAS Institute).

## 3 RESULTS AND DISCUSSION

### 3.1 Rice characterization

**FIGURE 1:**
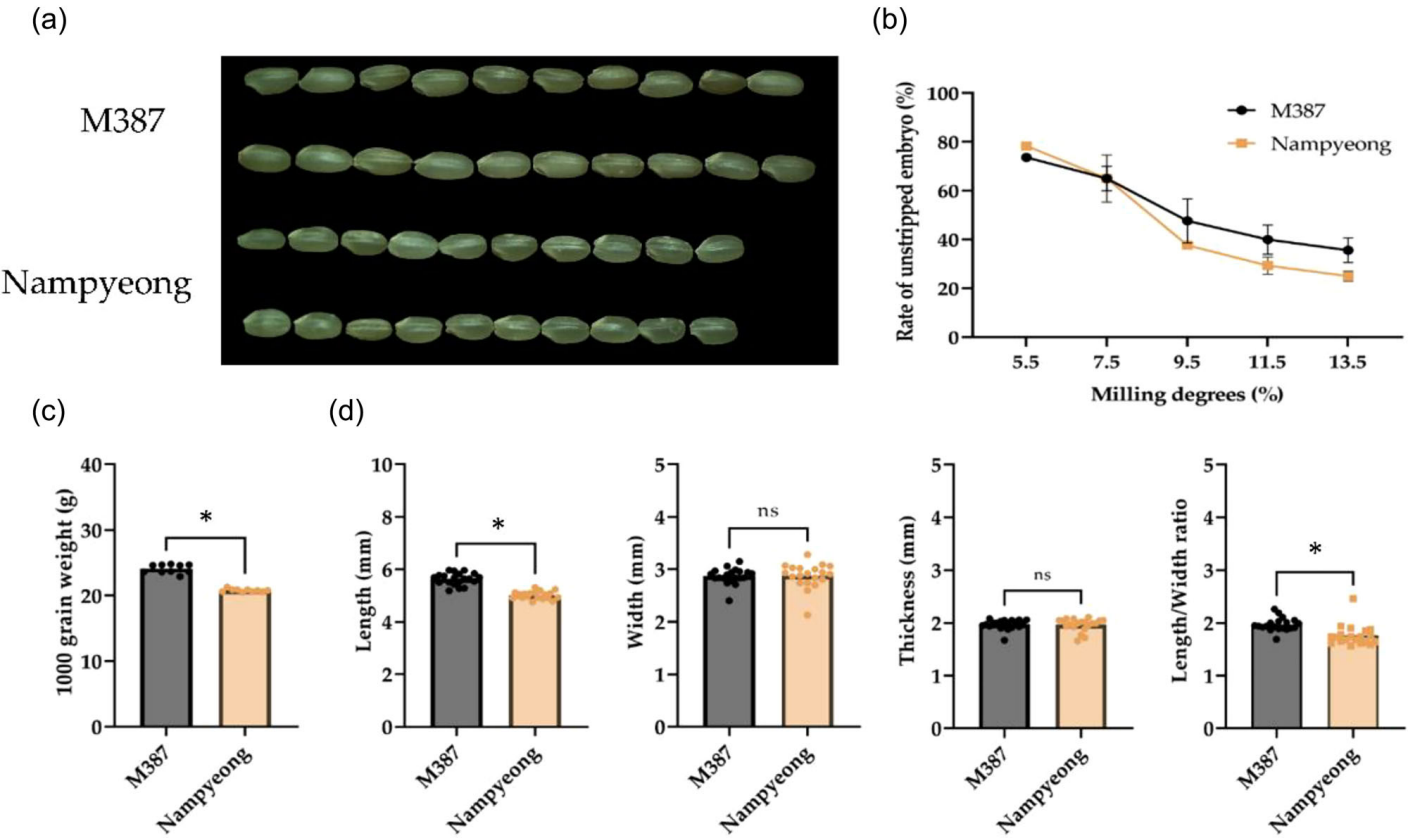
**Characterization of “M387” and “Nampyeong” rice**. (a) Image of 10 grains of brown rice. (b) Changes in unstripped embryo rate according to milling degrees of brown rice. (c) Thousand‐grain weight of brown rice. Values are presented as mean ± standard deviation (*n* = 10). Asterisks denote a significant difference between groups (*p < 0.05). (d) Average grain length, width, thickness, and length/width ratio of brown rice. Values are presented as mean ± standard deviation (*n* = 20). Asterisks denote a significant difference between groups (*p < 0.05), and “ns” indicate non‐significant differences.

The morphological traits of the semi‐waxy rice (8%–12% amylose content) (Liu et al., 2021) variety “M387” and the Japonica rice variety “Nampyeong” (18%–20% amylose content) were compared to assess their suitability for milling and consumption (Figure 1). One of the key parameters analyzed was the unstripped embryo rate, an important indicator of nutritional quality, as the embryo is rich in essential nutrients, including vitamins, minerals, and dietary fiber. Retaining the embryo during milling is desirable from a nutritional standpoint but can also affect the appearance and quality of the final milled rice product.

The results showed that the unstripped embryo rate decreased remarkably as the DOM increased for both varieties. At a milling degree of 5.5%, “M387” retained 73.7% of its embryo, whereas “Nampyeong” retained 78.3%. This initial difference highlights the slightly better embryo retention of “Nampyeong” at the lowest milling degree, which could be attributed to differences in grain structure or hardness. However, as the milling degree increased to 13.5%, the unstripped embryo rate dropped more dramatically in “Nampyeong,” reaching 25.0%, whereas “M387” retained 35.7% of its embryo (Figure 1b). This suggests that “M387” may have a more durable embryo structure or is less affected by milling pressure, which could result in better retention of nutritional components during processing.

The importance of the unstripped embryo rate lies in its direct impact on the nutritional value of rice. The embryo contains a high concentration of essential nutrients such as vitamin E, GABA, and other bioactive compounds. Consequently, the higher unstripped embryo rate of “M387” at higher milling degrees may offer advantages in terms of nutritional quality compared with those of “Nampyeong.”

The thousand‐grain weight of the two varieties also differed considerably. “M387” had a higher thousand‐grain weight than that of “Nampyeong” across all milling degrees. At a milling degree of 5.5%, the thousand‐grain weight of “M387” was 24.7 g, compared with 23.4 g for “Nampyeong” (Figure 1c,d). This suggests that “M387” grains are denser or larger than those of “Nampyeong,” which could influence both processing characteristics and consumer preferences. Larger, denser grains are often associated with higher milling yields and may be preferred by consumers due to their appearance and ease of handling during cooking.

In addition to grain weight, grain dimensions were analyzed, including length, width, and the length‐to‐width ratio. “M387” showed significantly greater grain length and width than those of “Nampyeong” (*p* < 0.001), confirming its larger overall grain size. However, the length‐to‐width ratio did not significantly differ between the two varieties, indicating that although “M387” grains are larger overall, their shape remains comparable to that of “Nampyeong.” The larger grain size of “M387” may contribute to its higher thousand‐grain weight and could be advantageous in milling, as larger grains often result in higher yields of milled rice and may be more visually appealing to consumers.

Another critical parameter analyzed was grain size and shape uniformity, which affects milling efficiency and final product quality. “M387” displayed a slightly more uniform grain size than that of “Nampyeong,” which could contribute to more consistent milling results and reduce the likelihood of breakage during processing. Uniform grains are preferred in the milling industry because they allow for even polishing and reduce the occurrence of broken rice, which can negatively impact final product value.

The higher weight and larger size of “M387” grains, coupled with its superior embryo retention at higher milling degrees, suggest that this variety may offer advantages in both processing and nutritional quality. These characteristics make “M387” a promising candidate for further development and optimization in milling processes, particularly for markets prioritizing nutritional content and visual quality. Moreover, the ability of “M387” to retain a higher percentage of the embryo during milling could position it as a valuable option for health‐conscious consumers who seek rice varieties with enhanced nutritional benefits, such as higher levels of GABA, vitamins, and antioxidants.

In conclusion, the rice characterization analysis demonstrates that “M387” possesses traits superior to those of “Nampyeong” in terms of grain size, weight, and embryo retention. These characteristics make “M387” ideal for optimizing milling techniques to balance nutritional quality with consumer preferences for appearance and texture.

### 3.2 Physicochemical properties

**FIGURE 2:**
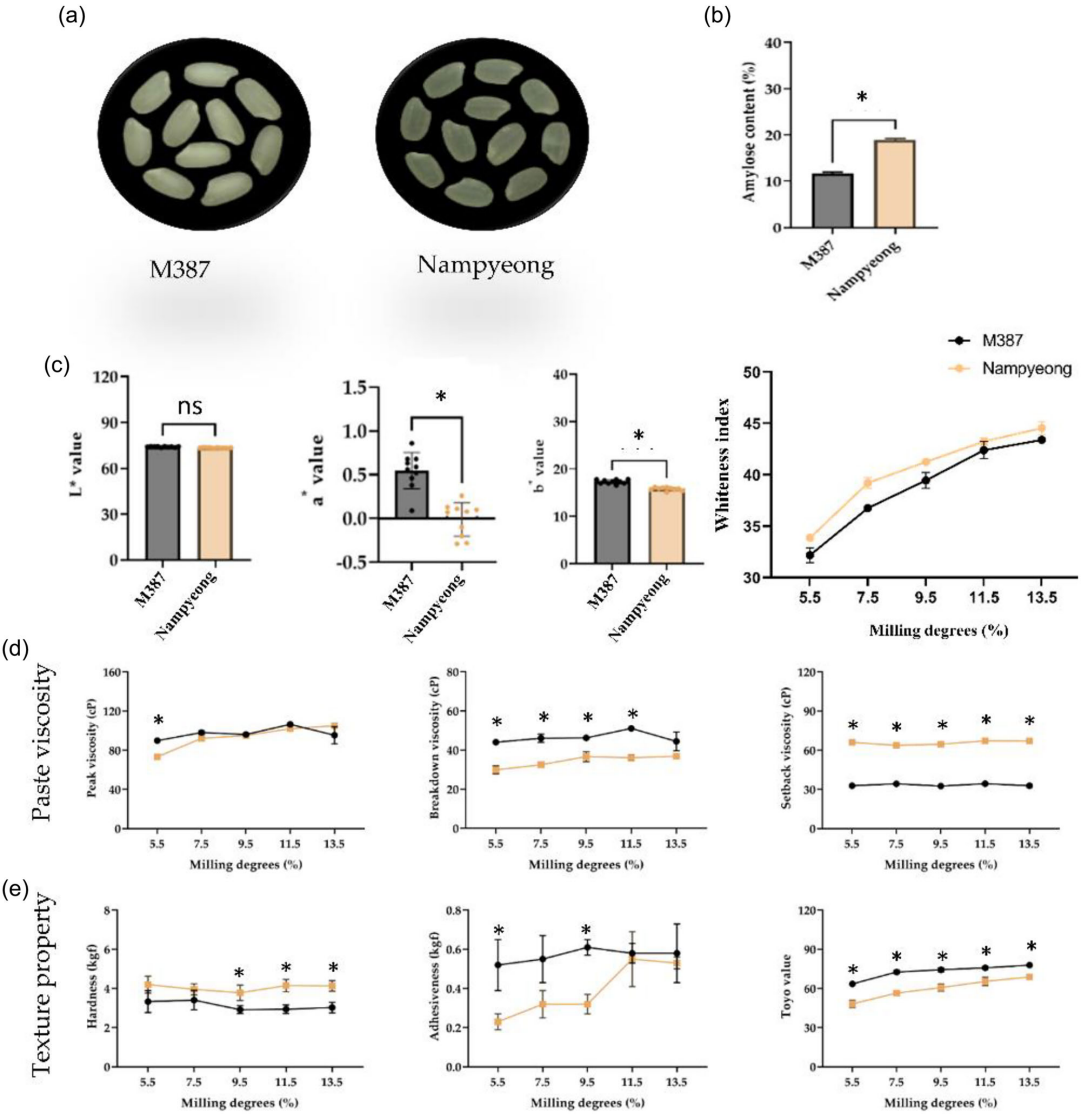
**Physicochemical properties of white “M387” and “Nampyeong’” rice**. (a) Image of white rice. Distribution of (b) amylose content, (c) color value, (d) paste viscosity (rapid visco analyzer), and (e) texture properties of cooked white rice. Values are presented as mean ± standard deviation (*n* = 20). Asterisks denote a significant difference between groups (*p < 0.05).

The physicochemical properties of “M387” and “Nampyeong” were evaluated across different milling degrees to understand how these properties impact rice quality in terms of nutrition, cooking performance, and consumer preferences (Figure 2). Key parameters assessed include moisture, amylose, protein content, color values, pasting properties, and texture analysis.

The moisture content of the rice samples was measured to ensure uniformity in milling. Rice with higher moisture content can be more difficult to mill as it may stick to the milling machine or result in inconsistent polishing. In this study, the moisture content remained relatively consistent across both varieties and milling degrees, ranging between 12% and 13% for both varieties. This uniformity ensured that other physicochemical characteristics were not affected by moisture variations, allowing for a more accurate comparison between the two varieties.

Rice varieties with higher amylose content tend to be firmer and less sticky, whereas those with lower amylose content, such as glutinous and semi‐waxy varieties, tend to be softer and stickier. “M387” had a consistently lower amylose content than “Nampyeong” at all milling degrees, aligning with the typical characteristics of semi‐waxy rice. At the reference milling degree of 9.5%, “M387” recorded an amylose content of 11.5%, whereas “Nampyeong” exhibited a higher value of 18.4% (Figure 2a,b). This lower amylose content in “M387” contributes to its softer texture when cooked, which is often preferred for dishes requiring softer, stickier rice.

In amylose content analysis, “M387” consistently maintained its lower amylose content relative to that of “Nampyeong,” reinforcing its suitability for consumers seeking softer and stickier rice varieties. In addition, “M387” exhibited lower amylose content than “Nampyeong,” contributing to a softer and stickier texture, which is desirable for culinary applications (Hebishy et al., 2024). The texture analysis further confirmed that the lower hardness and adhesiveness of “M387” make it preferable for consumers seeking softer rice varieties. These results are consistent with existing literature on the relationship between amylose content and rice texture, where lower amylose levels are associated with increased stickiness and softer mouthfeel (Liu et al., 2021, 2022; Yao et al., 2020; Zhang et al., 2021).

The visual appeal of rice is a significant factor influencing consumer preferences, and the color of milled rice is often used as an indicator of its quality. The color values of “M387” and “Nampyeong” were measured using the CIELAB system, which evaluates L*, a*, and b*. Higher L* values indicate whiter rice (Figure 2c). These results may correspond to the pasting properties, which are influenced by the amylose and amylopectin content of starch.

The pasting properties of rice, as measured by a rapid visco analyzer, provide insights into how rice behaves during cooking and starch gelatinization. Key parameters, such as peak, breakdown, and setback viscosity, were measured for both varieties across different milling degrees. Interestingly, at a milling degree of 9.5%, there was no significant difference in RVA peak viscosity between “M387” (95.05 cP) and “Nampyeong” (96.11 cP). This suggests that despite differences in starch composition between the two varieties, their pasting behavior under these milling conditions remains comparable (Figure 2d).

The breakdown viscosity, which measures starch stability during cooking, was lower in “Nampyeong” relative to that in “M387.” The setback viscosity, which reflects the degree of starch retrogradation (hardening) after cooling, was similarly lower in “M387,” suggesting that it remains softer even after cooling, making it ideal for dishes served at room temperature or cold.

The texture of cooked rice is a critical factor that directly influences consumer satisfaction. Hardness and adhesiveness were measured using a texture analyzer to quantify the differences in texture between “M387” and “Nampyeong” across different milling degrees.

From 9.5% to 13.5% of milling degree, “M387” showed lower hardness values compared with those of “Nampyeong,” particularly at higher milling degrees. At 9.5% milling, the hardness of “M387” was 3.17 kgf, whereas that of “Nampyeong” was higher at 3.98 kgf (Figure 2e). This lower hardness value of “M387” indicates a softer texture. As the DOM increased, the hardness of both varieties decreased, but “M387” maintained its softer texture relative to that of “Nampyeong.” In addition, adhesiveness, which measures the stickiness of the rice, was also lower in “M387” from 5.5% to 9.5% of milling degree though both varieties showed increased adhesiveness at higher milling degrees (Figure 2e).

The color of milled rice, evaluated through CIELAB parameters (L*, a*, and b*), was further analyzed using the WI to provide a more comprehensive understanding of the visual appeal of the samples. The WI, a derived metric combining L*, a*, and b* values, showed between milling degrees and rice varieties (Figure 2c). For both “M387” and “Nampyeong,” the WI increased as the milling degree rose from 5.5% to 9.5%, reflecting the removal of bran and germ layers that contribute to discoloration.

The Toyo value, a quantitative measure glossiness of rice, was assessed across different milling degrees for both “M387” and “Nampyeong” (Figure 2e). The results revealed a strong effect of milling degree on the Toyo values of both varieties. At lower milling degrees (5.5% and 7.5%), the Toyo values were lower, reflecting the influence of residual bran and germ, which can impact the texture negatively. Conversely, at higher milling degrees (9.5%–11.5%), the Toyo values improved, peaking at 9.5% for both varieties and maintaining a plateau thereafter. For “M387,” the Toyo taste value reached 74.3 at 9.5% milling, compared with 60.7 for “Nampyeong.” These findings indicate that the optimal milling degree for maximizing glossiness aligns with the 9.5% identified for maximizing palatability.

### 3.3 Antioxidant capacity

**FIGURE 3:**
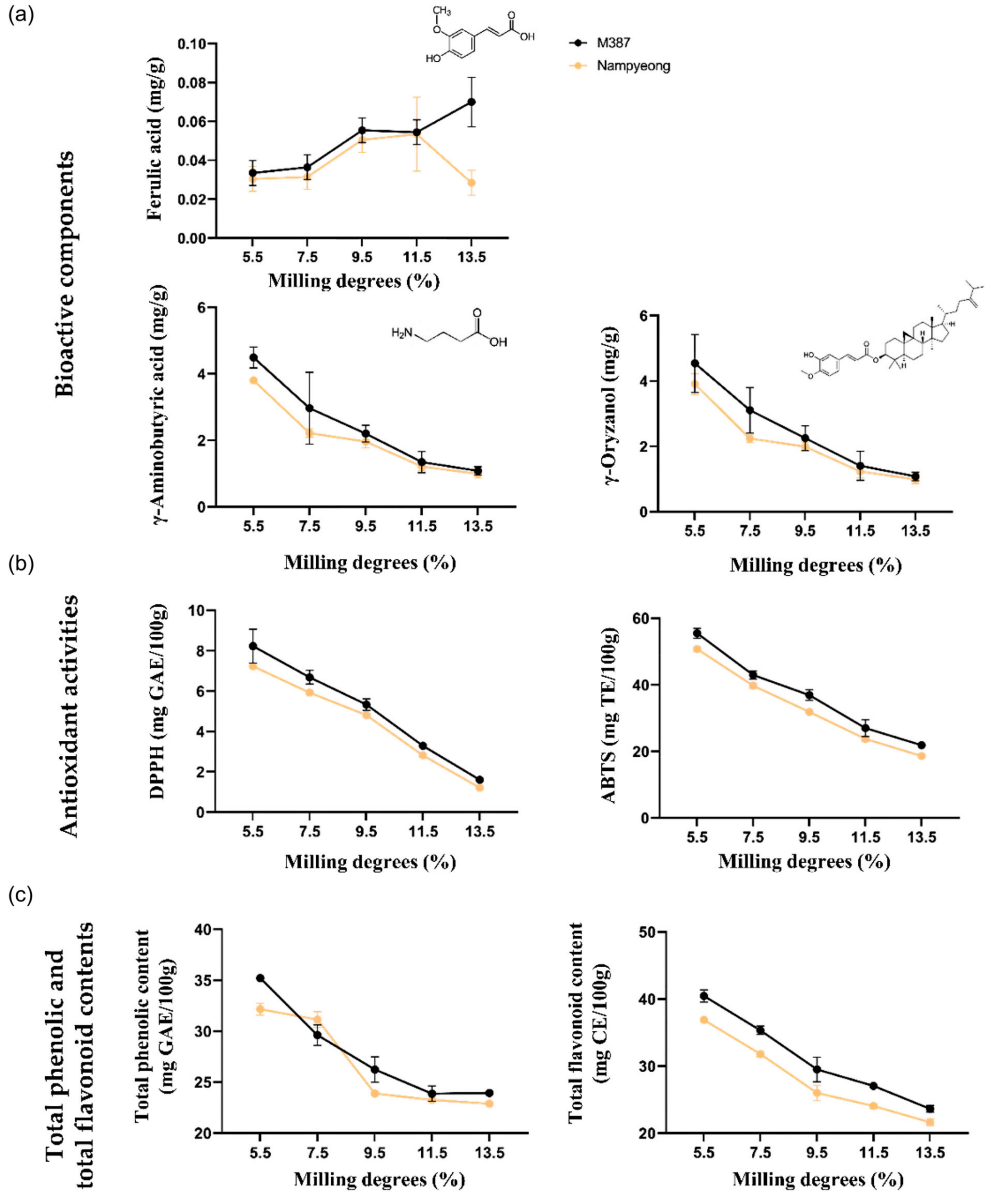
**Antioxidant capacity of white “M387” and “Nampyeong” rice**. (a) Ferulic acid, γ‐aminobutyric acid (GABA), and γ‐oryzanol content. (b) DPPH and ABTS equivalent antioxidant capacity. (c) Total phenolic acid and total flavonoid content. Values are presented as mean ± standard deviation (*n* = 3).

**FIGURE 4:**
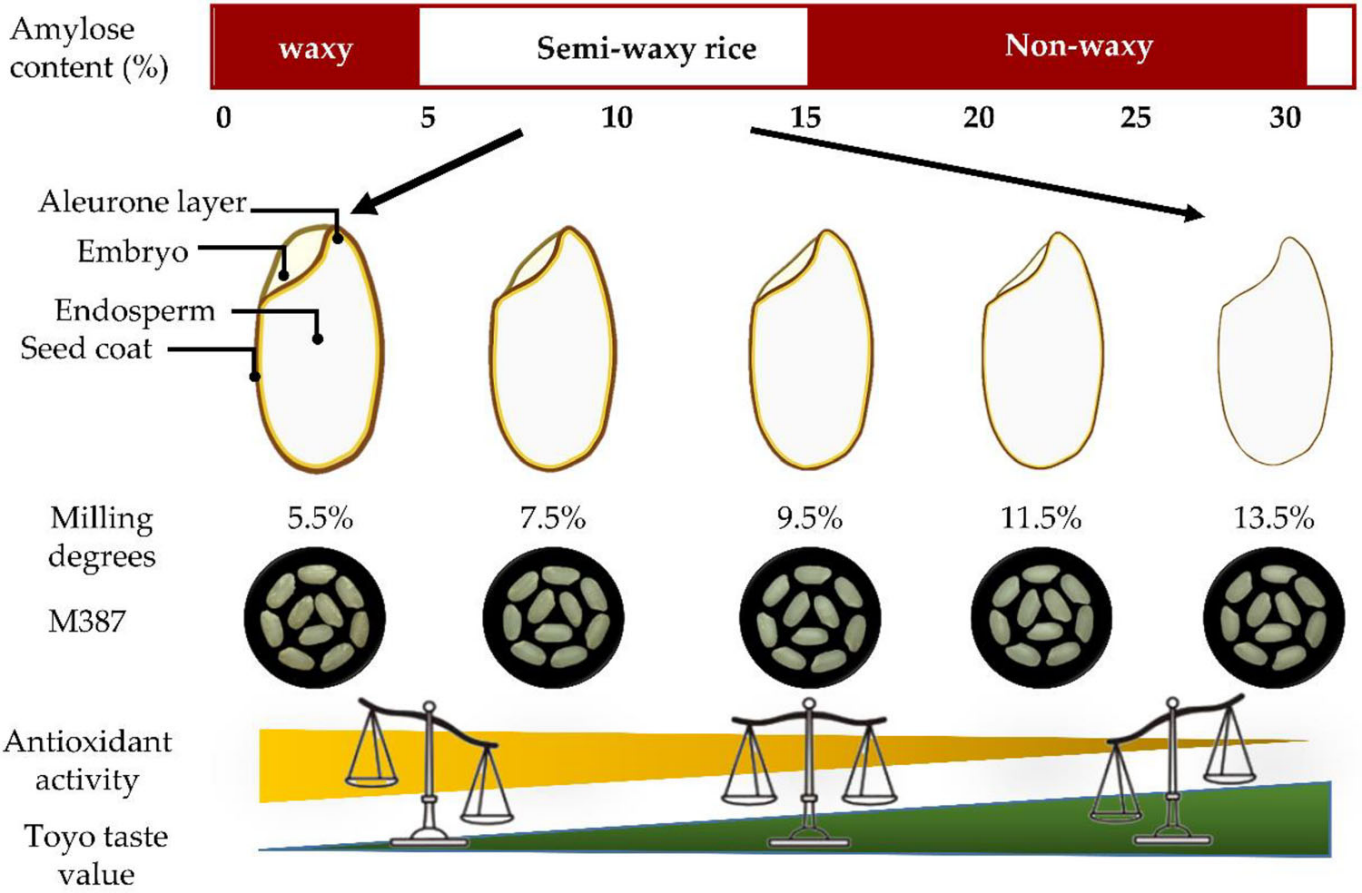
Schematic model depicting optimization of milling degree in relation to antioxidant content and eating quality.

The antioxidant capacities of “M387” and “Nampyeong” were assessed to determine the impact of milling degree on the retention of key bioactive compounds that contribute to the health benefits of rice (Figure 3). The primary antioxidants analyzed in this study included GABA, ferulic acid, γ‐oryzanol, and total phenolics and flavonoids, all of which play critical roles in reducing oxidative stress and providing various health benefits (Dudonne et al., 2009; Dykes & Rooney, 2007; Goufo & Trindade, 2017; Nishimura et al., 2016; Peanparkdee & Iwamoto, 2019; Sharif et al., 2014; Verma & Srivastav, 2020; Walter & Marchesan, 2011). In addition, DPPH and ABTS assays were performed to evaluate the radical scavenging activity of the rice varieties, reflecting their ability to neutralize free radicals. However, during the polishing process, most of the nutritional compounds in rice bran are lost (Kim et al., 2020; Liu et al., 2022; Peanparkdee & Iwamoto, 2019; Sharif et al., 2014). In this study, we analyzed antioxidant capacity to optimize palatability while aligning with health‐beneficial compounds (Figure S1‐S3). Figure 4

The results indicate that both variety and milling degree significantly influenced the retention of major antioxidants, including GABA, γ‐oryzanol, ferulic acid, total phenolic content, total flavonoid content, and radical scavenging activity (DPPH and ABTS assays). A clear trend was observed, where higher milling degrees resulted in a progressive reduction of all antioxidants due to the removal of the bran and germ layers, which are rich in these bioactive compounds. Among the two varieties, “M387” retained significantly higher antioxidant levels than “Nampyeong” across all milling degrees, suggesting that it has a greater capacity for preserving functional compounds.

The two‐way ANOVA results confirmed that both variety and milling degree had statistically significant effects (*p* < 0.05) on all antioxidant parameters analyzed. Specifically, a significant interaction effect (*p* < 0.05) was observed between variety and milling degree, indicating that the rate of GABA reduction varied depending on the variety. “M387” consistently retained higher GABA levels than “Nampyeong,” even at higher milling degrees, making it a more promising candidate for functional rice products. γ‐Oryzanol showed significant main effects of both variety and milling degree (*p* < 0.05), with a notable interaction effect. “M387” retained more γ‐oryzanol than “Nampyeong” at all milling levels, reinforcing its nutritional advantage. Ferulic acid content (*p* < 0.05), with levels increasing as milling increased. However, no significant interaction was observed from at 13.5% of milling degree, suggesting that both varieties followed a similar reduction pattern. Total phenolic content and total flavonoid content were significantly affected by variety and milling degree (*p* < 0.05), with “M387” showing greater retention. A significant interaction effect (*p* < 0.05) further indicated that milling had a stronger impact on antioxidant reduction in “Nampyeong.” A significant interaction effect (*p* < 0.05) was found, indicating that “M387” maintained higher free radical scavenging activity (DPPH and ABTS) than “Nampyeong,” particularly at lower milling degrees. In addition, to better understand these losses, a correlation analysis was conducted between milling degree and antioxidant content. A strong negative correlation was observed for all compounds (*ρ* > −0.85), confirming that increased milling degrees are associated with substantial reductions in bioactive compound content.

The results further highlight the need for controlled milling practices to balance nutrient retention (Figure 4). The trends observed in this study emphasize the need for balancing milling practices to retain nutritional and functional properties. The strong negative correlation between antioxidant content and milling degree reinforces the necessity of minimizing over‐polishing. “M387,” with its superior retention of antioxidants, emerges as a promising candidate for markets prioritizing health‐conscious products. By integrating results from this study with existing knowledge of bioactive compounds’ health benefits, we provide a comprehensive framework for optimizing milling degrees to enhance both the nutritional and taste qualities of rice.

## 4 CONCLUSION

Our findings demonstrate that although varietal differences influence bioactive compound content, the DOM remains a critical factor in optimizing functional properties and cooking quality. The analysis confirmed that milling progressively reduces the levels of key bioactive compounds within each variety, necessitating careful selection of milling parameters. The optimal milling degree of 9.5% for “M387” provides a valuable balance between nutrient retention and textural characteristics, making it a suitable candidate for both health‐conscious consumers and commercial applications. These insights highlight the importance of controlled milling processes in enhancing the nutritional value of rice, supporting the development of processing strategies that maximize both health benefits and marketability.

## AUTHOR CONTRIBUTIONS

**Youngho Kwon**: Conceptualization; methodology; writing—original draft; writing—review and editing. **Gi‐Un Seong**: Methodology; formal analysis; data curation; conceptualization; investigation. **Ju‐Won Kang**: Investigation. **So‐Myeong Lee**: Software. **Jin‐Kyung Cha**: Investigation. **Hyunjin Park**: Investigation. **Byungjun Jin**: Investigation. **Youngeun Lee**: Investigation. **Su‐Min Jo**: Validation. **Woo‐Jae Kim**: Investigation. **Seong‐Gyu Jang**: Investigation. **Jun‐Hyeon Cho**: Investigation. **Dong‐Soo Park**: Formal analysis. **Jong‐Hee Lee**: Conceptualization; methodology; writing—review and editing; funding acquisition; supervision.

## CONFLICT OF INTEREST STATEMENT

The authors declare no conflicts of interest.

## Supporting information



Figure S1 Analysis of ferulic acid in standards and “M387” at different milling degree.
Figure S2 Analysis of γ‐oryzanol in standards and “M387” at different milling degree.
Figure S3 Analysis of GABA in standards and “M387” at different milling degree.
