# Supplementary material for: Optimizing the degree of milling semi‐waxy rice variety to enhance its functional properties and cooking quality
Source: J Food Sci. 2025 Mar 20;90(3):e70142. doi: 10.1111/1750-3841.70142 (PMC11924880; doi:10.1111/1750-3841.70142)
Supplement: Supplementary file 1 — Figure S1 Analysis of ferulic acid in standards and “M387” at different milling degree. Figure S2 Analysis of γ‐oryzanol in standards and “M387” at different milling degree. Figure S3 Analysis of GABA in standards and “M387” at different milling degree. [file JFDS-90-0-s001.docx]

**Supplementary figure 1. Analysis of ferulic acid in standards and M387 at different milling degree.**





A, Calibration curve of standard. B, HPLC chromatogram of standard. C~G, HPLC chromatogram of M387 at 5.5, 7.5 9.5 11.5 13.5% of milling degree respectively.

**
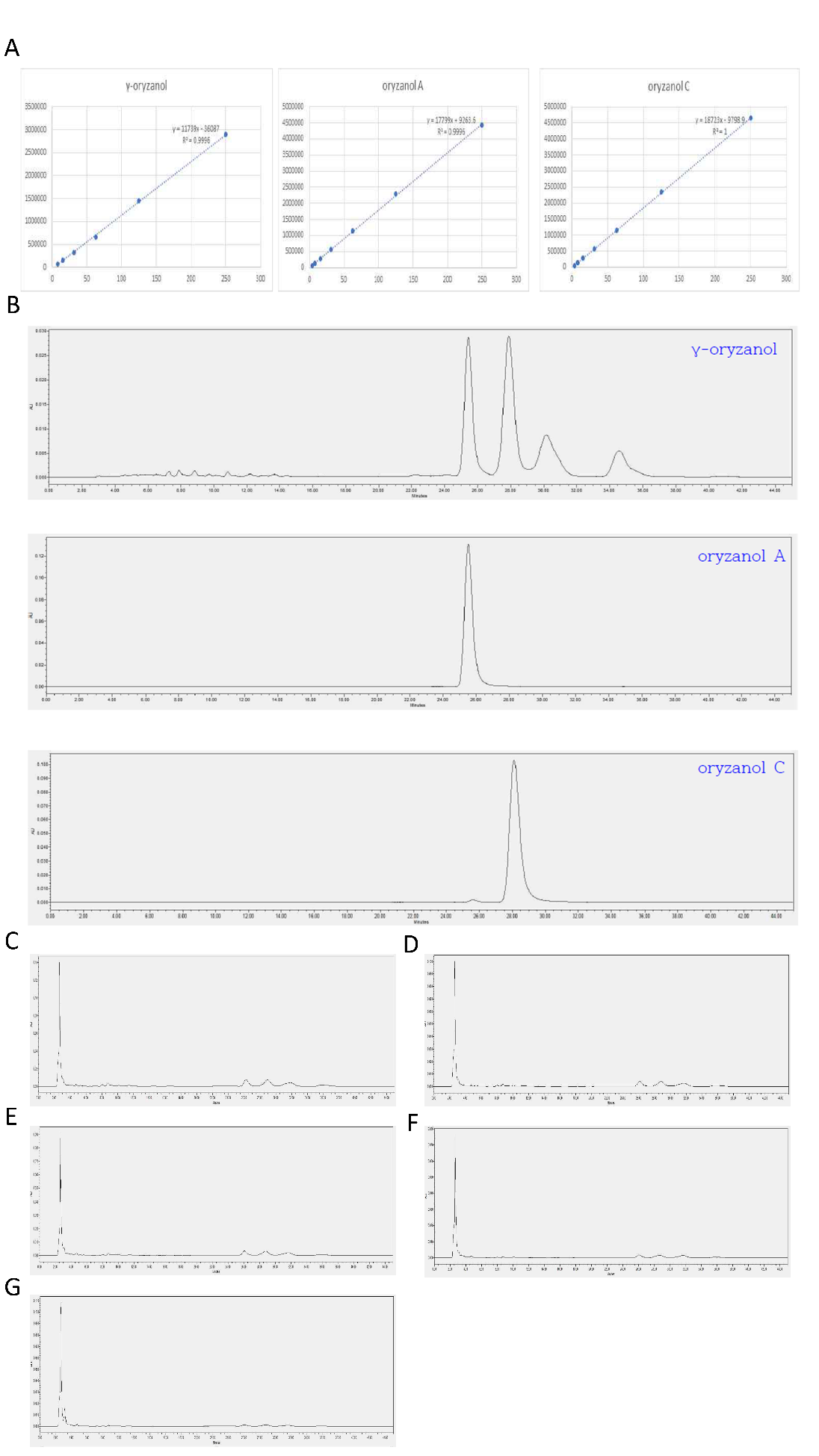
**

**Supplementary figure 2. Analysis of γ-oryzanol in standards and M387 at different milling degree.**

A, Calibration curve of standard of γ-oryzanol, oyzanol A and C. B, HPLC chromatogram of standard. C~G, HPLC chromatogram of M387 at 5.5, 7.5 9.5 11.5 13.5% of milling degree respectively.


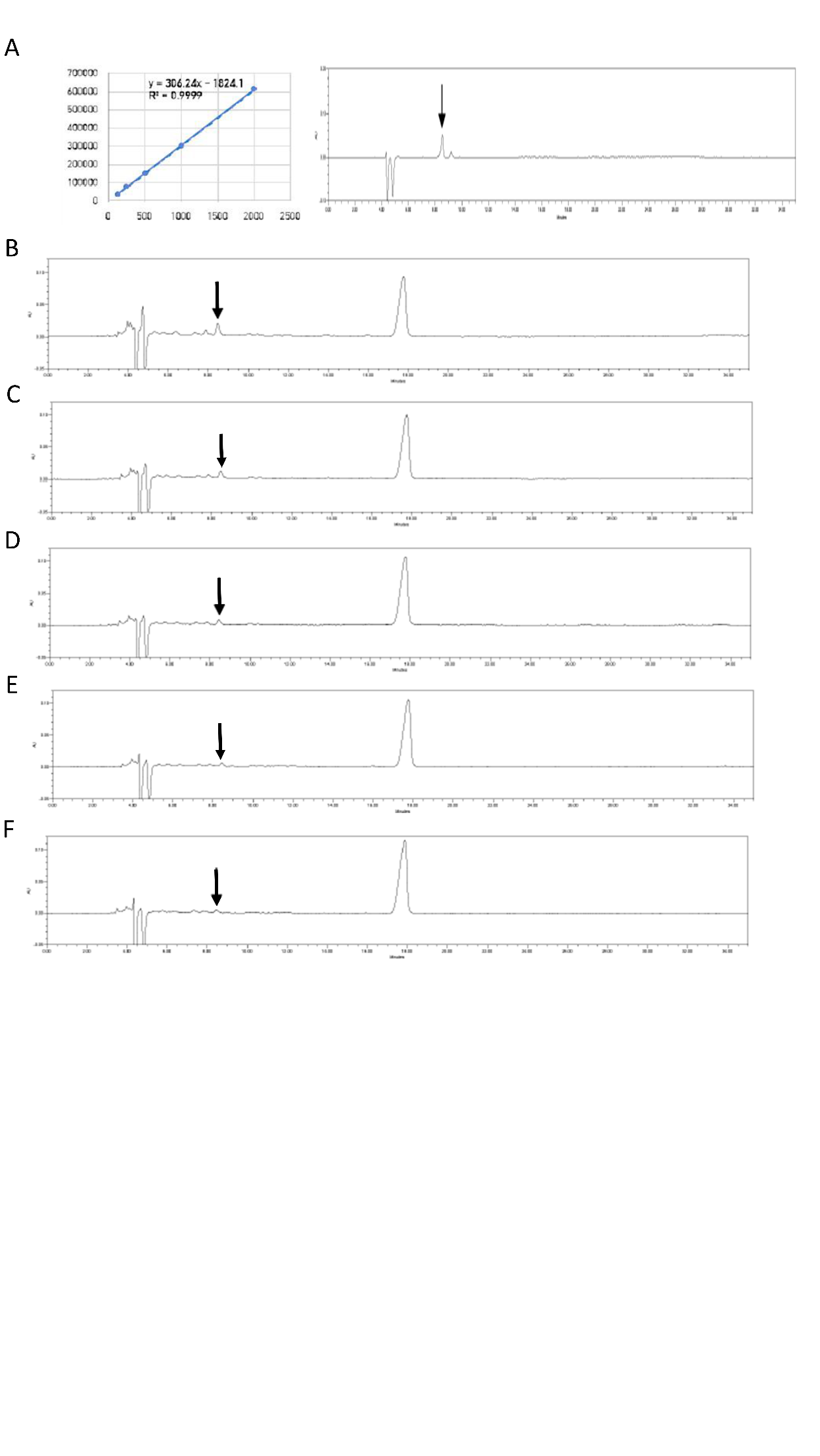


**Supplementary figure 3. Analysis of GABA in standards and M387 at different milling degree.**

A, Calibration curve of standard of GABA. B, HPLC chromatogram of standard. B~F, HPLC chromatogram of M387 at 5.5, 7.5 9.5 11.5 13.5% of milling degree respectively
